# Environmental exposures and adverse pregnancy outcomes in Ethiopia: A systematic review and meta-analysis

**DOI:** 10.1371/journal.pone.0288240

**Published:** 2023-07-12

**Authors:** Habtamu Demelash Enyew, Bethlehem Getachew Bogale, Abebe Beyene Hailu, Seid Tiku Mereta

**Affiliations:** 1 Department of Public Health, College of Health Sciences, Debre Tabor University, Debre Tabor, Ethiopia; 2 Department of Midwifery, College of Health Sciences, Madawalabu University, Robe, Ethiopia; 3 Department of Environmental Health Science and Technology, Jimma University Institution of Health, Jimma, Ethiopia; KMCH Institute of Health Sciences and Research, INDIA

## Abstract

**Background:**

Maternal exposures to environmental hazards during pregnancy are key determinants of birth outcomes that affect health, cognitive and economic status later in life. In Ethiopia, various epidemiological evidences have suggested associations between environmental exposures such as household air pollution, cigarette smoking, and pesticide exposure and pregnancy outcomes such as low birth weight, preterm birth, and birth defects.

**Objective:**

This review aimed at generating summarized evidence on the association between maternal exposure to environmental factors (household air pollution, cigarette smoking, and pesticide) and pregnancy outcomes (birth weight, preterm birth, and birth defects) in Ethiopia.

**Method:**

A systematic literature search was performed using PubMed, Google Scholar, and the Cochrane Library databases. All observational study designs were eligible for inclusion in the review. Quality assessment was conducted using the Newcastle-Ottawa Scale (NOS) quality assessment tools adopted for case-control and cross-sectional studies. The random-effects model was applied in computing the pooled estimates and their corresponding 95% confidence interval (CI). Funnel and Doi plots were used for detecting the potential publication bias. All statistical analyses were performed using comprehensive meta-analysis (CMA 2.0) and MetaXL version 5.3 software.

**Result:**

The pooled estimates revealed that prenatal biomass fuel use increased the risk of giving a low birth weight baby by twofold (OR = 2.10, 95% CI: 1.33–3.31), and has no separate kitchen increases the risk of having low birth weight baby nearly by two and half times (OR = 2.48, 95% CI: 1.25–4.92). Overall, using biomass fuel as the main energy source for cooking and /or having no separate kitchen from the main house is 2.37 times more likely to give low birth weight babies (OR = 2.37, 95% CI: 1.58–3.53). Active cigarette smoker women were 4 times (OR = 4.11, 95% CI: 2.82–5.89) more prone to have low birth weight babies than nonsmokers; and passive smoker women were 2.6 times (OR = 2.63, 95% CI: 1.09–6.35) more risked to give low birth weight babies. It was also estimated that active cigarette smoker women were nearly 4 times (OR = 3.90, 95% CI: 2.36–6.45) more likely to give preterm birth babies. Pesticide exposure during pregnancy also increases the risk of the birth defect 4 times (OR = 4.44, 95% CI: 2.61–7.57) compared with non-exposed pregnant women.

**Conclusion:**

Household air pollution from biomass fuel use, active and passive cigarette smoking, and pesticide exposures are significantly associated environmental risk factors for low birth weight, preterm birth, and birth defects in Ethiopia. Therefore, Pregnant and lactating women should be aware of these environmental hazards during pregnancy. Promoting clean energy and improved and efficient stoves at the household level will help to reduce household air pollution-related adverse health effects.

**Trial registration:**

PROSPERO 2022: CRD42022337140.

## Introduction

The pregnancy period is a crucial determinant of an infant’s health and survival for years to come [1, 2] which has been conceptualized as the developmental origin of health and disease (DoHaD) [3]. Because, it is a critical period of development when the developing organ systems of the fetus can be more vulnerable due to higher rates of cell division or changing metabolic capabilities [1, 4, 5].

Prenatal exposure to various classes of chemicals, including household air pollution, cigarette smoking, and pesticide exposure evidenced to have an association with adverse pregnancy outcomes [6–9]. Stillbirth, intrauterine growth retardation, low birth weight, congenital anomalies, neural tube defects, and impaired growth in the first years of life are some of the known visible adverse pregnancy outcomes due to various environmental exposures during pregnancy [1, 10, 11]. Various environmental exposures may also cause invisible changes in certain biological functions and can increase the risk of disease and dysfunction later in life [1, 2, 12, 13].

Though maternal characteristics and obstetric practices play an important role in contributing to adverse birth outcomes, several environmental risk factors have been studied for possible roles in influencing pregnancies and resulting in adverse birth outcomes which have a significant impact on mortality, morbidity, and healthcare costs [1, 2, 4, 14]. The changing lifestyle both in urban and rural communities exacerbates the widespread use of different chemicals in different human activities that puts pregnant women at high risk of these environmental exposures [4, 11, 15].

In Ethiopia, the evidence on the association between environmental pollution and adverse pregnancy outcomes is particularly strong for low birth weight, preterm birth, and birth defects with exposure to household air pollution [6, 16, 17], active and passive prenatal cigarette smoking [18–20] and pesticide use for agricultural activities [21–23]. Based on further analysis results of Ethiopian demography and health survey from 2000 to 2016, small birth weight prevalence is increasing from 7 to 13.2 percent [24].

It is also common to see Ethiopian women engaged in different agricultural, industrial, and other activities until their late pregnancy period which led to maternal exposure to chemicals in the workplace before and during pregnancy [25, 26]. Because they plant food for their families, cook meals, buy everyday products, wash clothes, and clean the house during which they or their family are exposed to various chemicals [27, 28]. Chemical pesticide uses in Ethiopia has been almost a threefold increase within a decade mainly due to the expansion of modern agricultural activities including commercial horticultural farms and small-scale irrigated farms [25, 29].

Though the body of scientific evidence links these environmental exposures with adverse pregnancy outcomes, the impacts have received less attention from clinicians compared with other maternal factors [12, 30]. The available evidences are also highly scattered and need systematic collecting from various epidemiological studies to obtain overall pooled summary estimates for an association between environmental exposures and pregnancy outcomes. Therefore, this systematic review and meta-analysis aimed at filling this gap by pooling relevant studies together to generate better evidence on the issue from which researchers and policymakers will be benefited.

### Objective

This paper aims to synthesize and generate the current pooled evidence from available epidemiological studies on environmental exposures and adverse pregnancy outcomes to facilitate further utilization of the evidences in Ethiopia.

## Methods

### Registration

This systematic review and meta-analysis was registered on PROSPERO with a protocol number CRD42022337140.

### Search strategies

**Fig 1:**
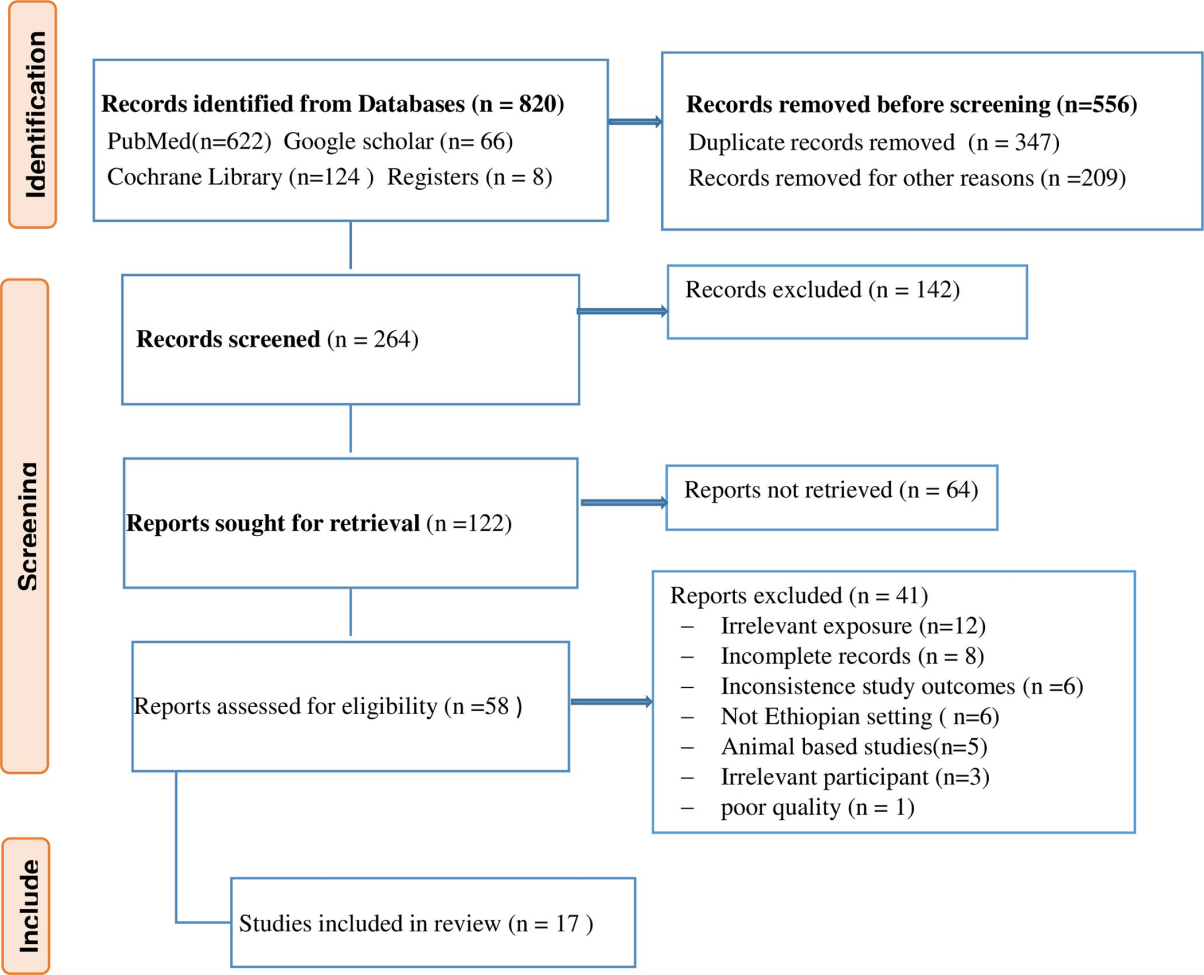
PRISMA 2020 flow diagram for new systematic reviews which included searches of databases and registers only.

Firsthand studies published in peer-reviewed journals were searched systematically using PubMed, Google Scholar, and the Cochrane Library databases. Repositories in Ethiopian universities were also visited for unpublished studies. Additional publications were identified based on references cited within the eligible studies. Internet (using the Google search engine) was also searched to maximize search efficiency. The above databases were searched following the new version of Preferred Reporting Items for Systematic Review and Meta-analysis (PRISMA) guideline for reporting protocol (Fig 1) and the PRISMA checklist to screen articles (S1 Checklist) [31]. Medical Subject Heading (MeSH) terms and free text words (Table 1) were used to identify relevant studies from the databases. The population, exposure, and outcome (PEO) terms were combined using the Boolean operator to search in the PubMed database.

**Table 1:** Search words.

Sources of chemicals	Exposure		Outcome measures	
Biomass fuel use	MeSH terms	Free text	MeSH terms	Free text
	• Indoor air pollution • Biofuel • biomass • Wood • Charcoal • cooking • coal	• Household air pollution • Domestic fuel • Kitchen smoke • Solid fuel • Firewood • crop residue • wood smoke	• Abortion • Miscarriage	• pregnancy outcomes • birth weight • Preterm birth • Stillbirth • premature birth • premature infant • small at gestational age
Cigarette smoking	• Smoking • Cigarette • Tobacco • substance	• Cigarette smoking • Tobacco smoking • Active smoker • Passive smoker • Env’tal tobacco smoking • substance use	• Abortion • Premature • Preterm • Prematurity • Small birth	• Low birth weight • Small at gestational age • Premature birth • Preterm birth
Use of pesticide	• Pesticide • Chemicals • Herbicides • Insecticides	• Pesticide exposure • Use of pesticide • Use of chemicals • Use of herbicides • Use of insecticides	• Birth defects	• Congenital anomalies • Neuro tube defects • Adverse birth outcomes

Boolean operators like “AND”, and “OR” were used to maximize the sensitivity and specificity of our search strategy by bringing key concepts together to identify environmental exposures and pregnancy outcomes search in the PubMed database. The improvement of the search terms was made while doing a trial among randomly selected articles and looking for other relevant terms within each concept from retrieved papers. After some rounds of trial and refinement of the search term, we formulate the final search term for PubMed as follows; (Pregnant women) OR (gravida)) OR (delivering women)) AND (indoor air pollution)) OR (biomass fuel use)) OR (solid fuel use)) OR (kitchen smoke)) OR (wood burning)) OR (household air pollution)) AND (cigarette smoking)) OR (passive smoker)) OR (active smoker)) OR (environmental tobacco smoker)) AND (pesticide exposure)) OR (herbicide use)) OR (use of insecticides)) OR (chemical use)) AND (pregnancy outcomes)) OR (low birth weight)) OR (preterm birth)) OR (stillbirth)) OR (congenital anomalies)) OR (birth defect)) OR (neural tube defect)) OR (small at gestational age)) AND (Ethiopia).

Since some databases do not support the use of Boolean or quotation, a standard search strategy is used only in PubMed, then later it is modified according to each specific database to get the best relevant results. Therefore, we modified the initial search terms for each database to get appreciated results. The detailed search strategy for each database is found in as S1 Table. Two reviewers (HD and BG) independently conducted the searches and assessed eligibility. The final selection of studies was based on consensus, with arbitration by a third reviewer (ST or AB) in the case of disagreement.

The search was built based on the population, exposure, and outcome (PEO) research question formulation strategy. Articles published up to May 30, 2022, from the multiple database searches were collated and duplicates were removed. All articles resulting from the databases were imported into one endnote library and duplicates were removed.

### Inclusion and exclusion

All observational studies (cross-sectional, case-control, and cohort) reported the association between environmental exposure (indoor air pollution, cigarette smoking, and pesticide exposure) and adverse pregnancy outcomes (low birth weight, preterm birth, and birth defects including neural tube defect) and conducted in Ethiopia were eligible for this systematic review and meta-analysis. Studies published Up to May 30, 2022, only in the English language were included mainly to avoid mistakes in the translation process. Other studies were excluded due to the following reasons: (a) articles that did not provide calculably or reported ORs and 95% CIs, (b) review studies, (c) none related to the above specified environmental exposures and pregnancy outcomes, and (d) animal or case studies.

### Risk of bias assessment

Three authors (HD, ST, and AB) independently assessed the risk of bias for each article using the Newcastle–Ottawa Scale (NOS) quality assessment tool (adapted for cross-sectional studies) [32]. This scale has three sections namely sample selection with maximum 5-star scores, comparability of the subjects in different outcomes with maximum 2-star scores, and outcome measurement with maximum 3-star scores. The risk of bias for case-control studies was assessed using Newcastle—Ottawa quality assessment tool for case-control and cohort studies [33]. This scale also has three similar sections (selection of cases rated maximum of 4-star scores, comparability of the case and control groups rated maximum of 2-star scores, and exposure measurement rated maximum of 4-star scores). In the NOS tool, authors rated each item using stars. An overall score was determined by adding all the items’ scores as stars equal one, while no stars equal zero. All studies were rated from a total of 10 scale scores and the result of each study against each criterion was reported as a separate supporting information in excel form (S1 Data).

### Data extraction

All the necessary data were extracted using a customized form based on the Cochrane Public Health Group data extraction form and cross-checked to avoid errors. The data extraction forms were designed to be tailored to the review questions. One author (HD) developed the first draft of the data extraction form to gather information on the items of interest. This was reviewed by ST and complemented and revised after discussion. Two authors (BG and AB) piloted the data extraction forms and extracted the information of interest from the included studies using the final version of the extraction form. One of the major modifications added after piloting the data extraction form was the formulation of the additional table (Table 3) containing the methodological approaches of the studies included.

The variables extracted were determined based on the review questions. Extracted data items were cross-checked between the reviewers, with any disagreement being resolved through consensus or via arbitration by a third reviewer if necessary (HD). The data extraction format was designed to include first author, publication year, study type, sample size, types of exposure, types of birth outcome (low birth weight and preterm birth), odds ratio with 95% CI and publication history (Table 2). In addition, study setting, exposure comparators, case ascertainment, confounder adjustment methods, and quality assessment scores were abstracted from each study (Table 3).

**Table 2:** Description of the included studies for computing the associated factors adverse pregnancy outcomes in Ethiopia.

Author, year	Study period	Study design	Region	Sample size	Outcome	Prevalence	Exposure	AOR (95% CI)	Publication history
Admasie et al., 2018 [16]	2017	Cross-sectional	SNNPR	1042	LBW	16.3	Biomass fuel use	3.83 (1.95–15.35)	Published
							NO separate kitchen	2.27(1.88–5.88)	
							No window	4.79 (1.56–14.69)	
							Cooking time > 3 hrs/d	2.45 (1.16–5.21)	
Demelash et al, 2015 [6]	2013	Case control	Oromia	383	LBW	-	Firewood fuel use	2.70 (1.01–7.17)	Published
							Kerosene fuel use	8.90 (2.54–31.11)	
							No separate kitchen	2.6 0(1.36–4.85)	
							Cow dung fuel use	14.4 (4.08–50.97)	
Kanno et al, 2021 [34]	2016	Cross-sectional	Nationwide	10,014	LBW	25.9	No separate kitchen	1.30 (1.10–1.60)	Published
Emebet D and Nigusie D, 2017 [7]	2014	Case-control	AA	347	LBW	-	Passive smoking	1.86 (1.08, 4.91)	Published
Tsegaye M & Yewbmirt S, 2020 [18]	2018–2019	Cross-sectional	SNNPR	472	LBW	34.1	Cigarette smoking	4.35 (2.46, 7.69)	Published
Alekaw Sema et al., 2019 [19]	2018	Cross-sectional	Dire Dawa	431	LBW	21	Cigarette smoking during pregnancy	3.97 (1.59, 9.88)	Published
Mingude et al, 2020 [20]	2018	Case-control	SNNPR	300	LBW		passive smoking	4.73 (1.42–15.7)	Published
Halil et al, 2019 [35]	2019	Cross-sectional	SNNPR	363	LBW	12.7	having a history of cigarette smoking	5.85 (1.18, 28.92)	Published
Bayew Kelkay et al, 2019 [36]	2018	Cross-sectional	Tigray	325	PTB	16.9	cigarette smoking	3.61(1.59–8.23)	Published
Deriba et al, 2021 [37]	2020	Case-control	Oromia	410	PTB		having a history of cigarette smoking	3.77 (1.35,10.56)	Published
Alekaw Sema et al, 2021 [38]	2019	Cross-sectional	Dire Dawa	412	PTB	9	Smoking during the current pregnancy	4.3, (1.29, 14.45)	Published
Taye et al.,2018 [39]	2018	case-control	AA and Amhara	414	CAS	-	Exposed to pesticides	9.96(1.23–80.19)	Published
Mekonnen et al, 2020 [21]	2018	Case-control	Oromia	409	CAS	-	Exposed to pesticides	3.19 (1.31, 10.96).	Published
Jemal et al, 2021 [40]		case-control	Oromia	418	CAS		Exposed to Pesticide	4.76 (1.57–14.47)	Published
Abebe et al, 2021 [22]	2016–2018	Case-control	Oromia	1138	CAS	-	Exposed to pesticides	3.92 (1.27, 12.18)	Published
Gashaw et al, 2021 [23]	2019–2020	Case-control	Amhara	243	NTD		exposed to Pesticides	5.34 (1.77, 16.05)	Published
Birhane et al. 2019 [41]		Case-control	Tigray	617	NTD		exposure to pesticide	5 (0.150–166.60)	Published
Atlaw et al. 2019 [42]		Case-control		462	NTD		exposure to pesticide	0.19 (0.02–2.21	Published

LBW: low birth weight; PTB: preterm birth; NTD: neural tube defect

**Table 3:** Additional methodological approaches of the studies included in this systematic review and meta-analysis.

Authors	Study Setting	Exposure Comparators	Case Ascertainment	Adjustment
Admasie et al.	Communities	• Biomass vs electricity • Cooking place inside house vs outside • No window vs > 3 windows • Improved stove vs open fire • Time spent cooking per day 3 hrs vs 1 hr	• Mothers recall	• Adjusted for wealth status, childbirth order, child spacing, mother and father education; sex of the child, number of windows, level of house ventilation; cooking place, time spent on cooking
Demelash et al.	Hospitals	• Using firewood vs electricity • Using kerosene vs electricity • Using cow dung vs electricity • House with no separate kitchen vs house with separate kitchen	• Measurement using a balanced Seca scale	• Adjusted for the birth interval, gravida, antenatal care (ANC) follow-up, gestational age at first ANC visit, deworming during pregnancy, maternal height, maternal weight, maternal BMI, history of pregnancy-related problems, history of alcohol drinking and khat chewing
Kanno et al.	Communities	• Kitchen location inside the house vs outdoor • Kitchen location in another building vs outdoor	• Mothers recall	Adjusted for • Child factor (gender of the baby and birth order} • Maternal characteristics (Age of the mother at first birth, maternal, educational status, BMI, Anemia level, maternal Chat Chewing and Alcohol drinking)
Emebet D & Nigusie D	Hospitals	• cigarette smokers vs nonsmokers	• Neonatal weighing scale	• Adjusted for weight gain, history of underweight, education, the income of the mother, and inter-pregnancy spacing
Tsegaye M & Yewbmirt S	Hospitals	• Smokers vs nonsmokers	• Digital Seca balance scale	• Adjusted for Maternal age, residency, educational status, occupation, marital status, birth interval, pregnancy type, ANC follow-up,
Alekaw Sema et al.	Hospitals	• Active smokers vs nonsmokers	• Balanced Seca scale	• Adjusted for residency, level of education, pregnancy intention, nutritional counseling, contraception use, pregnancy complications, ANC follow-up, mode of delivery, the height of the mother, and gestational age
Mingude et al.	Hospitals	• Husband smoking vs nonsmoking	Standard weighing scale	• Adjusted for marital status, monthly income, parity, history of preterm birth, maternal Rh factor, ANC visit, time start ANC visit, number of ANC visits, gestational age, types of gestation, birth interval, nutritional counseling, iron and folic acid supplementation, MUAC, anemia, history of chronic illness, maternal smoking status, chat chewing, alcohol drinking, and husband smoking status
Bayew Kelkay et al.	Hospitals	Cigarette smokers vs nonsmokers	Mothers’ recall	• Adjusted for medication intake during the most recent pregnancy, history of hyperemesis, previous abortion, previous stillbirth, previous preterm birth, previous cesarean delivery, history of vaginal bleeding, history of diagnosed urinary tract infection, previous malaria attack, previous multiple deliveries
Deriba et al,	Hospitals	cigarette Smokers vs nonsmokers	ANC records (either LNMP or early ultrasound)	Educational level, family size, history of alcohol drinking and adverse birth outcome, hemoglobin, current medical illness, ANC visit, and pregnancy status
Alekaw Sema et al,	Hospitals	Smokers vs nonsmokers	Balanced Seca scale	Residency, level of education, pregnancy intention, nutritional counseling, contraception use, pregnancy complications, ANC follow-up, mode of delivery, the height of the mother, and gestational age
Mekonnen et al.	Hospitals	Pesticide user vs non user	Medical examination	Women’s occupation, residence, number of infants, have been drinking alcohol and khat chewing
Abebe et al.	Hospitals	Pesticide user vs non user	Observation	Folic acid use, drinking alcohol, cigarette smoking, x ray exposure, DM, maternal illness, use of antibiotics, drug use in first 3 months and 4 to 6 months, caffeine intake, khat chewing, asthma, hypertensive disorders of pregnancy, ANC follow up
Gashaw et al.	Hospitals	Pesticide users vs nonusers	Gross visual examination and ultrasonography	maternal residence, no formal paternal education, unplanned pregnancy, preconception illness (fever), taking any drug during preconception, history of NTDs
Kelelaw et al.	Hospitals	Smokers vs nonsmokers	Either LNMP or 1^st^-trimester ultrasound	Parity, medication intake, hyperemesis gravida, history of abortion, stillbirth, preterm, hospital admission malaria and vaginal bleeding, hemoglobin level, and number of ANC visits
		Tobacco smoking partner vs nonsmoker partner		Weight gain, history of being underweight, education, the income of the mother, inter-pregnancy spacing,
Atlaw et al,	Hospitals	Passive smoker vs nonsmoker	Mother’s recall	Adjusted for • Mother age, religion, consanguinity, drug use during pregnancy, exposure to chemicals and toxins, history of abortion, family history of NTD, ANC follow-up, folic acid supplementation, and weight of the mother

### Outcome measurement

The outcome of this review is to determine the association between prenatal exposure to environmental risks (household air pollution, active and passive cigarette smoking, and pesticide exposure) and adverse pregnancy outcomes (low birth weight, preterm birth, and birth defects) as these are the most reported variables. Household air pollution exposure was assessed qualitatively based on the types of fuel used in the households, the location of the kitchen from the main house, the presence of windows, cooking frequency, and time spent in the kitchen. The comparison was made between low-pollution fuels (Electricity, liquefied petroleum gas (LPG), and biogas) and high-pollution fuels (charcoal, wood, crops, straw, and animal dung). Whereas cigarette smoking and pesticide exposures were assessed based on the self-reported response of the respondents about their exposure statuses an active or passive smoker or nonsmoker and pesticide user or nonuser. The pooled odds ratio with its 95% CI was calculated based on outcomes from the primary studies. All exposure and outcomes ascertainment techniques for each study are reported in a separate table (Table 3).

### Data processing and analysis

All relevant extracted data were analyzed using comprehensive meta-analysis (CMA 2.0) and MetaXL version 5.3 software. Heterogeneity among included studies was assessed using Cochran’s Q test and I^2^ statistics with its p-value. As heterogeneity between studies in design, geographic location, exposure to different biomass fuels, different outcome measurement methods, different confounding factors, and methodological differences are expected, a random-effects meta-analysis model was used to compute the pooled estimate. The standard errors of the studies were plotted to detect asymmetry in the distribution of studies.

In addition, the Doi plot (a symmetrical mountain-like plot) with Luis Furuya-Kanamori (LFK) index was used to assess publication bias. Because the Doi plot is more visible than the funnel plot to observe the symmetricity of the two areas of the graph and the LFK index is more sensitive than Egger’s regression to detect publication bias. LFK index <1 indicates no asymmetry, between 1 and 2 suggests minor asymmetry, and index >2 suggests major asymmetry [43]. Pooled odds ratio with 95% confidence intervals was presented in the forest plot format.

## Results

Initially, we identified 820 studies in our search. But only 17 (10 case-control and 7 cross-sectional) studies with 18,560 mother-infant pair participants were selected for data extraction as summarized in the PRISMA flowchart (Fig 1). Three studies (two cross-sectional and one case-control) reported the association between biomass fuel use and low birth weight. These papers have also stated the presence of an association between types of kitchens (separated or non-separated from the main house) and low birth weight. The pooled estimate was done for both reports. Seven (five cross-sectional and two case-control) studies analyzed the association between cigarette smoking and low birth weight. Additional seven case-control studies reported the presence of an association between pesticide exposure and birth defects.

### Household air pollution and low birth weight

Low birth weight infants are defined as weighing less than 2,500 grams at birth [44]. Birth weight and length of pregnancy are the two important predictors of neonatal and infant health [45, 46]. Exposure to biomass smoke inside the home from cooking with solid fuels adversely affects pregnancy outcomes [8, 47, 48] whereas more than 93 percent of households in Ethiopia use some type of solid fuel for cooking which is a known source of indoor air pollution [49]. In this paper, we computed the pooled odds ratio (OR) of biomass fuel use and low birth weight from three eligible papers [6, 16, 34]. Accordingly, infants born from mothers using biomass fuel were twice more likely to be born with low birth weight than infants born from clean fuel user mothers (OR = 2.10, 95% CI: 1.33–3.31).

**Fig 2:**
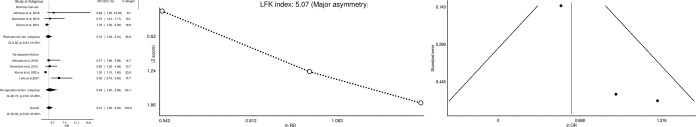
a: Forest plot of the pooled odds ratio (OR) and 95% confidence interval (CI) of the association between household solid biomass fuel use and low birth weight in Ethiopia. b: DOI plot of the pooled odds ratio (OR) of the association between household solid biomass fuel use and low birth weight in Ethiopia. c: Funnel plot of the pooled odds ratio (OR) of the associations between household solid biomass fuel use and low birth weight in Ethiopia.

Sensitivity analysis was performed step-by-step leave-one-out method to assess the impact of each primary study on the pooled OR and to detect the possible causes of heterogeneity between studies. After the exclusion of the highest OR 3.83, the pooled value reduced to 1.76 (95% CI: 1.34–2.31, p = 0.37), and with the additional exclusion of the lowest OR study, 1.70 the pooled value became 3.19 (95% CI: 1.57–6.49, p = 0.63). This indicates no significant outlier from the studies. In addition, Doi plots were used for detecting potential publication bias. The plot was asymmetrical with (LFK index = 5.07), suggesting major asymmetry. Accordingly, the results indicated the presence of potential publication bias (Fig 2B and 2C).

As cooking behavior is an important factor, it was considered in this review and the above studies were pooled to generate the association between cooking in a non-separated kitchen and low birth weight. The pooled odds ratio showed that infants from mothers who were cooking in the non-separated kitchen in the main house were nearly twice more likely to give birth with low birth weight babies than those who were cooking in separated kitchens from the main house (OR = 2.48, 95% CI: 1.25–4.92). The overall odds of giving low birth weight babies among biomass fuel users and/or having no separate kitchen for cooking was estimated to be (OR = 2.37 (95% CI: 1.59–3.53) (Fig 2A).

### Exposure to cigarette smoke and low birth weight

**Fig 3:**
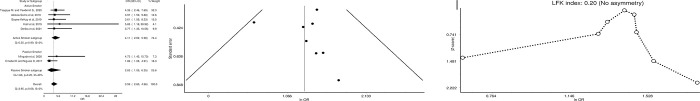
a: Forest plot of the pooled odds ratio (OR) and 95% confidence intervals (CIs) of the associations between cigarette smoking and low birth weight in Ethiopia. b: Doi plot of the pooled odds ratio (OR) of the associations between cigarette smoking and low birth weight in Ethiopia. c: Doi plot of the pooled odds ratio (OR) of the associations between cigarette smoking and low birth weight in Ethiopia.

It is also evidenced that infants born to active or passive cigarette smoker mothers weigh substantially less [9, 50] and are more likely to be preterm birth [36, 37, 51] than infants born to non-smoker women. In this review, we identified seven eligible studies that reported the association between active cigarette smoking [18, 19, 35–37] and passive or secondhand cigarette smoking [7, 20] and low birth weight in Ethiopia. The pooled estimate revealed that active cigarette smoker women are 4 times more likely to give low birth weight babies than nonsmoker women (OR = 4.11, 95% CI: 2.82–5.98). Whereas inhalation of particles from cigarette smoke during passive smoking has a twofold more risk to give low birth weight baby (OR = 2.63, 95% CI: 1.09–6.35). The overall pooled estimate of seven studies showed that inhalation of cigarette smoke actively and /or passively during pregnancy has 3.6 times more prone to give low birth weight babies than non-smokers (OR = 3.59, 95% CI: 2.60–4.96) (Fig 3A).

The sensitivity of each study was checked to identify smaller or larger reported odds ratios that could affect the pooled estimate by varying instability. But, the sensitivity analysis of this review showed no study has significantly affected the pooled result ranging from (OR = 3.28, 95%CI: 2.21–4.86) to (OR = 4.16, 95%CI: 2.90–5.95). To detect the potential publication bias for this group of studies, the Funnel and Doi plots were used. As a result, the asymmetry funnel plot suggested publication bias exists among these included articles (Fig 3B). This means studies with non-significant results may not have been published, which leads to the possibility that the pooled effect size may be overestimated. But, the Doi plot with (LFK index = 0.20), suggests no asymmetry (Fig 3C).

### Exposure to cigarette smoke and preterm birth

**Fig 4:**
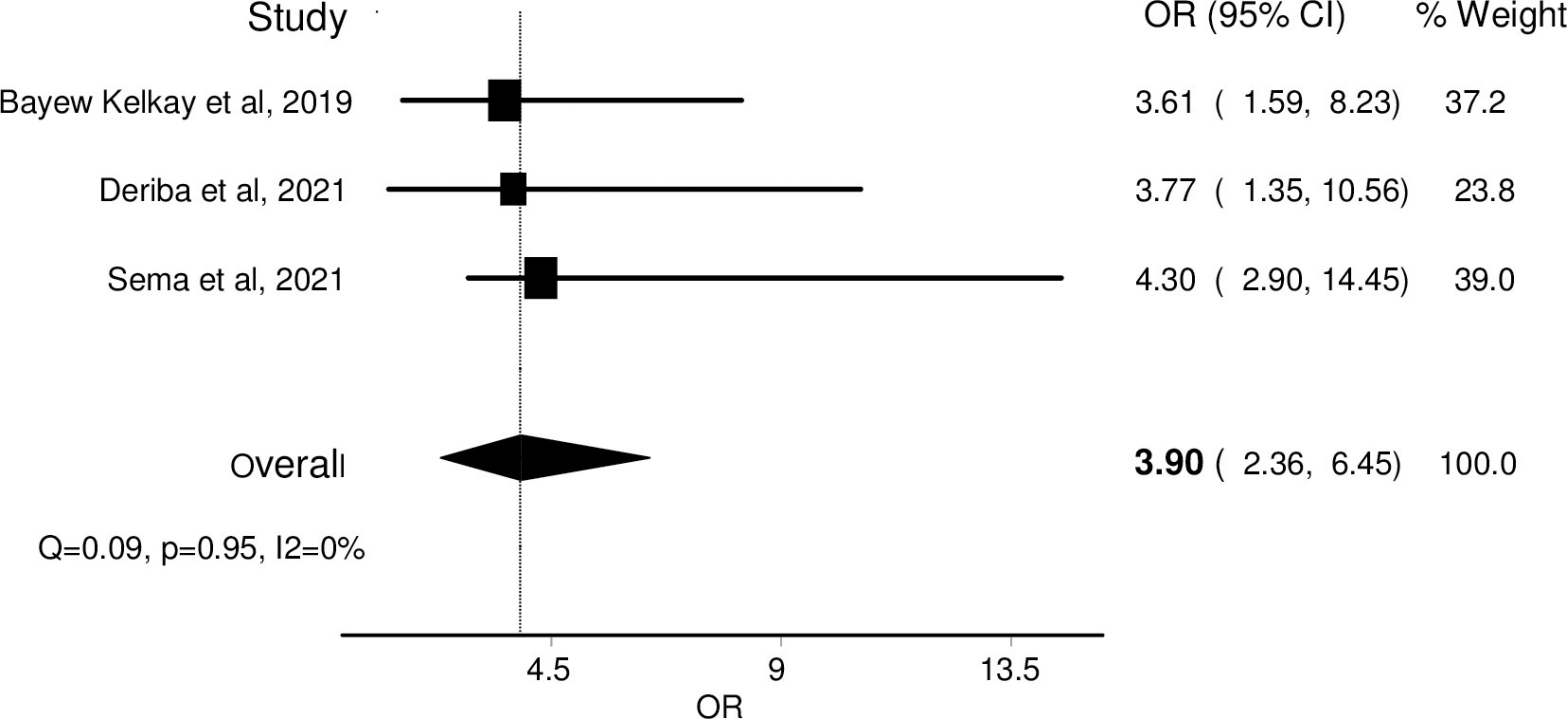
Forest plot showing the association between cigarette smoking and preterm birth in Ethiopia.

Preterm birth is a live birth before 37 completed weeks of gestation [52]. Three studies [19, 36, 37] reported the association between prenatal active cigarette smoking and preterm birth in Ethiopia. The ORs from individual studies were combined and the pooled estimate was computed and telling as women who were exposed to cigarette smoke during their pregnancy period were nearly four times more likely to give preterm babies (OR = 3.90, 95% CI: 3.36–6.45) (Fig 4).

### Pesticides and birth defects

**Fig 5:**
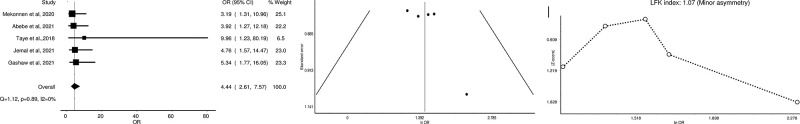
a: Forest plot showing association between pesticide exposure and birth defect in Ethiopia. b: Funnel plot showing association between pesticide exposure and birth defect in Ethiopia. c: Doi plot showing association between pesticide exposure and birth defect in Ethiopia.

Suggestions are also growing and revealing that exposure to pesticides during pregnancy increased the risk of certain types of birth defects [25, 27, 53]. This review tried to assess pesticide exposure as one environmental exposure and its association with the occurrence of birth defects using five eligible papers [21–23, 40, 54] with 2,622 infant-mother pairs/subjects. The individual ORs were extracted with the highest OR estimate reported by Gashaw et al. (OR = 5.34, 95% CI: 1.77–16.05). The pooled estimate (OR = 4.44, 95% CI: 2.61–7.57) shows the existence of a statistically significant association between pesticide exposure and birth defects with the interpretation of mothers with pesticide exposure were 4 times more likely to give birth with babies having a certain birth defect than mothers with no pesticide exposure during pregnancy (Fig 5A).

The Doi plot with (LFK = 1.07) demonstrates the presence of minor symmetry with apriori concern about negative publication bias (studies showing a negative effect of pesticides more likely to be published) (Fig 5B). In contrast, there was some degree of subjectivity with the funnel plot due to the unclear scattering of the points on the plot (Fig 5C).

## Discussion

This study was conducted to examine the association between environmental exposure during pregnancy and pregnancy outcomes in Ethiopia. Environmental exposures included in this review are biomass fuel use for cooking, heating, and lighting, active and passive cigarette smoking, and exposure to pesticides. The pooled odds ratio in this review indicated that prenatal exposure to air pollution from biomass fuel increases the risk of giving a low birth weight baby by twofold.

The result is in agreement with a growing body of both epidemiological and toxicological research that links household air pollution from biomass fuel use with increased risks of low birth weight and preterm birth [55–58]. The result is also supported by other systematic reviews and meta-analysis studies done in sub-Saharan Africa [8] and worldwide[59]. Similarly, nationwide studies in Bangladesh and Zambia reported that the combustion of solid biomass fuels at home increases the risk of low birth weight and preterm birth [56, 60]. ln Malawi, an association has observed a reduction in birth weight in infants born to mothers that use biomass fuels [61]. The risk of adverse outcomes in offspring due to perinatal exposure to cooking biomass fuels was investigated by Mi-Sun Lee and colleagues and reported increased odds of low birth weight and a significant reduction in the gestational age [62]

The possible plausible mechanism for the observed association can be explained in different ways hypothesized by different researchers [63, 64]. One plausible is that pollution particles from cooking smoke may move across the membranes of the lungs and be carried to other parts of the body, affecting placental function and the fetus [65]. Another potential mechanism is that pollutants may initiate systemic inflammation or oxidative stress that affects the health of both the pregnant woman and her baby [58].

Currently, it seems to be evidenced that maternal exposure to cigarette smoke (both active and passive) during pregnancy contributes to adverse pregnancy outcomes including a decrease in birth weight [9, 50, 66, 67]. In this review, maternal exposure to cigarette smoke during pregnancy was associated with low birth weight showing that low birth weight infants were four and three times as likely to be born to active and passive cigarette smoker mothers compared to those born to nonsmoker mothers respectively. Even the pooled OR for cigarette smoking was larger in magnitude compared with the results from biomass fuel smoke. The possible reasons may be inhalation of cigarette smoke during active or passive smoking, the particles are relatively concentrated, resulting in relatively high-risk increases for reproductive and infant health outcomes.[50, 66]. The association between cigarette smoking during pregnancy and low birth weight is also well established in different systematic review and meta-analysis studies [68–70]. As stated in Gómez-Roig et al., smoking is associated with placental abnormalities, preterm birth, stillbirth, or impaired growth and development, as well as intellectual impairment later in life [1]. Based on another large-scale case-control study in Jordan, neonates from active smoking mothers had significantly lower birth weights compared to neonates from Passive and nonsmoking women [9].

In this review, the pooled OR was computed from three studies [19, 36, 37] that reported the association between cigarette smoking and preterm birth (PTB). The estimated summary OR showed cigarette smoking during pregnancy was nearly 4 times more likely to give PTB compared to their unexposed counterparts. Similar evidence is reported by another systematic review and meta-analysis study [71]. The possible plausibility is that harmful nicotine and other chemicals in cigarettes have effects on human germ cells and are known to have adverse effects on the development of the embryo, especially during organogenesis [72].

Though scientific evidence is surplus on the influence of multiple medical and socioeconomic statuses of pregnant women on birth defects, it is also suspected to be associated with exposure to pesticides. Wide-ranging research worldwide has identified potential associations between in-utero pesticide exposure and several adverse birth outcomes [73]. This study also shows the significant association between exposure to pesticides and birth defects in Ethiopia. Accordingly, it was found that women with a history of pesticide exposure during pregnancy were 4 times more likely to give birth to newborns with birth defects than those with no history of exposure to a pesticide. The result is in agreement with another systematic review conducted at the continental level in Africa, where mothers having pesticide exposure were 3 times more likely to have newborns with neural tube defects [74]. Maternal exposure to pesticides was significantly associated with birth defects as reported in another systematic review conducted in South Africa [75]. It revealed that women exposed to the pesticide were three times more likely to have newborns with neural tube defects than women who were not exposed to the pesticide [11]. Similarly, a report by Nieuwenhuijsen et al. from a summary of meta-analyses of epidemiological studies revealed that occupational exposure to pesticides and solvents is significantly associated with some congenital anomalies [2].

### Strength and limitation

Our effort to maximize the search efficiency during retrieving all available relevant literature could be mentioned as the main strength of this paper. But, the number of original studies conducted on environmental exposure and pregnancy outcomes is very limited in Ethiopia. This may potentially affect the magnitude of pooled estimated values. The way the selected studies measured the exposure status and outcome variables differed, which potentially has an impact on the summary results due to misclassifications. Some confounding factors, such as the type of cooking stoves, personal exposure, and the availability of windows or chimneys also remained uncontrolled in some studies. In addition, since this review is based on the available observational (case-control and cross-sectional) studies, all the methodological and statistical limitations of the primary studies have an impact on the quality of this systematic review and meta-analysis work. Especially, recall and selection bias cannot be eliminated. Since the number of studies included in this systematic review and meta-analysis work is relatively small (< 20), the Chi-squared statistical test (Q test) and the I^2^ test for heterogeneity should be interpreted very cautiously.

## Conclusion

Household air pollution from biomass fuel use, active and passive cigarette smoking, and pesticide exposure is associated with environmental risk factors for low birth weight and birth defects in Ethiopia. Pregnant women living in households that utilize biomass fuel or are exposed to cigarette smoke were more at risk of giving low birthweight babies than mothers who live in households that utilizes clean energy sources or non-cigarette smokers. In this review. it is also evidenced that pesticide exposure has a positive association with birth defects including neuro tube defects. Therefore, Pregnant and lactating women should be aware of the risks of indoor air pollution, pesticide exposure, cigarette smoking, and another environmental risk factor during their pregnancy period. Promoting clean and effective solid fuels and improved and efficient stoves at the household level will help to reduce household air pollution-related adverse health effects, specifically low birth weight. Future studies should pay more attention to effective exposure measurement including personal and biomarker sampling for different environmental pollutants during the whole gestation period.

### The implication of the study

Adverse pregnancy outcomes including low birthweight, preterm birth, neural tube defect, and congenital anomalies are significant public health problems in Ethiopia. Because of Exposure of women to various environmental chemicals (mainly indoor air pollution due to biomass fuel use, active and passive tobacco smoking, and use of pesticides during agricultural activities) before and during pregnancy leads women to inhale them for hours which consequently leads to adverse pregnancy outcomes. Despite the progress made to reduce the burden of maternal and infant mortality and morbidity in the country, the magnitude of these adverse pregnancy outcomes is still one of the challenges that need to be addressed. Identifying the association between biomass fuel use, cigarette smoking, and pesticide use with pregnancy outcomes can help the efforts to revise, amend or implement treatment guidelines for the clinician and contribute valuable imputes for policy and decision-makers.

## Supporting information

S1 Checklist PRISMA_2020_checklist. (DOCX)

S1 Data Risk of bias assessment result. (XLS)

S2 Data (CSV)

S1 Table Manipulation guides for online database searches. (DOCX)

S2 Table Detailed search strategy for twelve database searches. (DOCX)

## Abbreviations

ANC: Antenatal care
CA: congenital anomalies
CI: Confidence interval
LBW: Low birth weight
NTD: Neural tube defect
OR: odds ratio
PTB: Preterm birth
