# Supplementary material for: Environmental exposures and adverse pregnancy outcomes in Ethiopia: A systematic review and meta-analysis
Source: PLoS One. 2023 Jul 12;18(7):e0288240. doi: 10.1371/journal.pone.0288240 (PMC10337917; doi:10.1371/journal.pone.0288240)
Supplement: S1 Checklist — (DOCX) [file pone.0288240.s001.docx]

| **Section and Topic** | **Item #** | **Checklist item** | **Location where item is reported** |
| --- | --- | --- | --- |
| **TITLE** | | |  |
| Title | 1 | Identify the report as a systematic review. | - Reported at the 1^st^ page of the manuscript |
| **ABSTRACT** | | |  |
| Abstract | 2 | See the PRISMA 2020 for Abstracts checklist. |  |
| **INTRODUCTION** | | |  |
| Rationale | 3 | Describe the rationale for the review in the context of existing knowledge. | - Reported at the last paragraph in the introduction section |
| Objectives | 4 | Provide an explicit statement of the objective(s) or question(s) the review addresses. | - Reported at page 3 from LN 72 -78 |
| **METHODS** | | |  |
| Eligibility criteria | 5 | Specify the inclusion and exclusion criteria for the review and how studies were grouped for the syntheses. | - Reported at page 5 LN 113 |
| Information sources | 6 | Specify all databases, registers, websites, organisations, reference lists and other sources searched or consulted to identify studies. Specify the date when each source was last searched or consulted. | - Reported at page 3 through page 4 LN 86. The last date for searching was may 30,2022 |
| Search strategy | 7 | Present the full search strategies for all databases, registers and websites, including any filters and limits used. | - Reported at page 3 through page 4 in detail |
| Selection process | 8 | Specify the methods used to decide whether a study met the inclusion criteria of the review, including how many reviewers screened each record and each report retrieved, whether they worked independently, and if applicable, details of automation tools used in the process. | - This issue is reported at page 5 through page 6 in detail. But we didn’t use automation tool in the process |
| Data collection process | 9 | Specify the methods used to collect data from reports, including how many reviewers collected data from each report, whether they worked independently, any processes for obtaining or confirming data from study investigators, and if applicable, details of automation tools used in the process. | - This important issue is sufficiently reported at page 6 and LN 136 |
| Data items | 10a | List and define all outcomes for which data were sought. Specify whether all results that were compatible with each outcome domain in each study were sought (e.g. for all measures, time points, analyses), and if not, the methods used to decide which results to collect. | - This one is detailed under the subtitle of outcome measurement on page 7 |
|  | 10b | List and define all other variables for which data were sought (e.g. participant and intervention characteristics, funding sources). Describe any assumptions made about any missing or unclear information. | - these all - - These all are stated in the table 3 on page 10 |
| Study risk of bias assessment | 11 | Specify the methods used to assess risk of bias in the included studies, including details of the tool(s) used, how many reviewers assessed each study and whether they worked independently, and if applicable, details of automation tools used in the process. | - The topic is detailed on page 5 under the subtitle assessment of risk of bias |
| Effect measures | 12 | Specify for each outcome the effect measure(s) (e.g. risk ratio, mean difference) used in the synthesis or presentation of results. | - The issue is stated in table 2on page 5 |
| Synthesis methods | 13a | Describe the processes used to decide which studies were eligible for each synthesis (e.g. tabulating the study intervention characteristics and comparing against the planned groups for each synthesis (item #5)). | - Stated under subtitle of data processing and analysis on page 9 |
|  | 13b | Describe any methods required to prepare the data for presentation or synthesis, such as handling of missing summary statistics, or data conversions. | - The data is simply collected/extracted from primary studies and pooled to present it using forest plot and other graphical method. The detail is on page 9 again |
|  | 13c | Describe any methods used to tabulate or visually display results of individual studies and syntheses. | - Reported in table 2 |
|  | 13d | Describe any methods used to synthesize results and provide a rationale for the choice(s). If meta-analysis was performed, describe the model(s), method(s) to identify the presence and extent of statistical heterogeneity, and software package(s) used. | - It is detailed on page 14 through 15 and 16 |
|  | 13e | Describe any methods used to explore possible causes of heterogeneity among study results (e.g. subgroup analysis, meta-regression). | - Well described on the same page 14 through 16 |
|  | 13f | Describe any sensitivity analyses conducted to assess robustness of the synthesized results. | - It is presented on page 14 and separate table is prepared to show the sensitivity one by one exclusionmethod |
| Reporting bias assessment | 14 | Describe any methods used to assess risk of bias due to missing results in a synthesis (arising from reporting biases). | - Risk of bias is assessed /page 5/ and presented as supplementary document |
| Certainty assessment | 15 | Describe any methods used to assess certainty (or confidence) in the body of evidence for an outcome. | - The 95% confidence interval and p-values were used to assess certainty on page 14 |
| **RESULTS** | | |  |
| Study selection | 16a | Describe the results of the search and selection process, from the number of records identified in the search to the number of studies included in the review, ideally using a flow diagram. | - Stated on page 14 and presented with PRIMA flow diagram as fig 1 |
|  | 16b | Cite studies that might appear to meet the inclusion criteria, but which were excluded, and explain why they were excluded. | - It is also presented in the PRISMA diagram with reasons of exclusion |
| Study characteristics | 17 | Cite each included study and present its characteristics. | - All included studies cited in table 2 and its charateterstic too |
| Risk of bias in studies | 18 | Present assessments of risk of bias for each included study. | - Reported on page 5 and presented as separate supplementary file |
| Results of individual studies | 19 | For all outcomes, present, for each study: (a) summary statistics for each group (where appropriate) and (b) an effect estimate and its precision (e.g. confidence/credible interval), ideally using structured tables or plots. | - Each study summary statistics presented in table 2 on page 7 through 8 and the pooled result presented using forest plot |
| Results of syntheses | 20a | For each synthesis, briefly summarise the characteristics and risk of bias among contributing studies. | - The characteristics of each study and its case ascertainment techniques presented on page 10 through 13 in table 3 |
|  | 20b | Present results of all statistical syntheses conducted. If meta-analysis was done, present for each the summary estimate and its precision (e.g. confidence/credible interval) and measures of statistical heterogeneity. If comparing groups, describe the direction of the effect. | - Related to heterogeneity of the studies presented using funnel and Doi plots |
|  | 20c | Present results of all investigations of possible causes of heterogeneity among study results. | - Lit is presented on page 9 and LN 16 |
|  | 20d | Present results of all sensitivity analyses conducted to assess the robustness of the synthesized results. | - Presented on page 14 |
| Reporting biases | 21 | Present assessments of risk of bias due to missing results (arising from reporting biases) for each synthesis assessed. | - All included studies evaluated using Newcastle–Ottawa Scale and the results presented as separate supplementary file |
| Certainty of evidence | 22 | Present assessments of certainty (or confidence) in the body of evidence for each outcome assessed. | - Presented in forest plots |
| **DISCUSSION** | | |  |
| Discussion | 23a | Provide a general interpretation of the results in the context of other evidence. | - The result interpretation and comparison with other studies is done throughout the discussion part on page 16 though 18 |
|  | 23b | Discuss any limitations of the evidence included in the review. | - Stated on page 18 |
|  | 23c | Discuss any limitations of the review processes used. | - Stated on page 18 |
|  | 23d | Discuss implications of the results for practice, policy, and future research. | - Reported on page 19 |
| **OTHER INFORMATION** | | |  |
| Registration and protocol | 24a | Provide registration information for the review, including register name and registration number, or state that the review was not registered. | - It is registered on PROSPERO with a protocol number CRD42022337140 and presented on page 3 and LN 84 |
|  | 24b | Indicate where the review protocol can be accessed, or state that a protocol was not prepared. | - The review protocol was prepared and can be accessed |
|  | 24c | Describe and explain any amendments to information provided at registration or in the protocol. | - No amendment has been taken place |
| Support | 25 | Describe sources of financial or non-financial support for the review, and the role of the funders or sponsors in the review. | - No financial support is given for this review |
| Competing interests | 26 | Declare any competing interests of review authors. | - No competing of interest among the review authors |
| Availability of data, code and other materials | 27 | Report which of the following are publicly available and where they can be found: template data collection forms; data extracted from included studies; data used for all analyses; analytic code; any other materials used in the review. | - These all can be accessed with request |
