# Supplementary material for: Environmental exposures and adverse pregnancy outcomes in Ethiopia: A systematic review and meta-analysis
Source: PLoS One. 2023 Jul 12;18(7):e0288240. doi: 10.1371/journal.pone.0288240 (PMC10337917; doi:10.1371/journal.pone.0288240)
Supplement: S1 Table — (DOCX) [file pone.0288240.s004.docx]

**Supplementary Table 1. Manipulation guides for online database searches**

| **No.** | **Databases (Total 3)** | **Explanation** |
| --- | --- | --- |
| **1** | **PubMed** | 1. Perform PubMed search. 2. Choose the reports that the reviewer wants to export (if reviewer does not choose any report, PubMed system will automatically select all).0 3. Click on Send To, choose File. 4. Choose MEDLINE format. 5. Click Create File. 6. Move PubMed file to a suitable directory. |
| **2** | **Cochrane** | 1. Perform Cochrane search. |
| **3** | **Google Scholar** | 1. Perform Google Scholar search. 2. Click on Settings. 3. At Results per page, change to 20. 4. At Bibliography manager, choose Show links to import citations into EndNote, then click Save. 5. On Navigation Toolbar in Google chrome browser, click on the Zotero icon. 6. Choose Select All in the drop down window, and then click OK. 7. Move to the next pages and perform the same task (step (5) and (6)). 8. At the bottom-right corner of the browser (on Add-on Bar), click on the Zotero 9. On the new interface, click on Actions button, choose Export library. 10. Choose RIS format. 11. Click OK. 12. Move the exported Google Scholar file to a suitable directory. |
