# Supplementary material for: Environmental exposures and adverse pregnancy outcomes in Ethiopia: A systematic review and meta-analysis
Source: PLoS One. 2023 Jul 12;18(7):e0288240. doi: 10.1371/journal.pone.0288240 (PMC10337917; doi:10.1371/journal.pone.0288240)
Supplement: S2 Table — (DOCX) [file pone.0288240.s005.docx]

Supplementary Table 1. Detailed search strategy for twelve database searches

| **no** | ***Databases (Total= 3)*** | **Search Terms** | **Results**  **Total = 820** |
| --- | --- | --- | --- |
| 1 | **PubMed** | (Pregnant women) OR (gravida)) OR (delivering women)) AND (indoor air pollution)) OR (biomass fuel use)) OR (solid fuel use)) OR (kitchen smoke)) OR (wood burning)) OR (household air pollution)) AND (cigarette smoking)) OR (passive smoker)) OR (active smoker)) OR (environmental tobacco smoker)) AND (pesticide exposure)) OR (herbicide use)) OR (use of insecticides)) OR (chemical use)) AND (pregnancy outcomes)) OR (low birth weight)) OR (preterm birth)) OR (still birth)) OR (congenital anomalies)) OR (birth defect)) OR (neural tube defect)) OR (small at gestational age)) AND (Ethiopia). | 622 |
| 2 | **Cochrane Library** | (biomass fuel OR household air pollution OR Cigarette smoking OR pesticide use) AND (low birth weight OR preterm birth OR birth defect) AND Ethiopia | 124 |
| 3 | **Google Scholar** | Where my words occur: in the title of the article: | 276 |
